# Shear wave elastography to distinguish between hard and soft mucin in pseudomyxoma peritonei

**DOI:** 10.1515/pp-2025-0044

**Published:** 2026-05-08

**Authors:** Carissa Vici, Nishka Tapaswi, Ashish Vaska, David L. Morris

**Affiliations:** Peritonectomy Unit, St George Hospital, South Eastern Sydney Local Health District, Kogarah, NSW, Australia; University of New South Wales Saint George and Sutherland Clinical Campuses, Kogarah, NSW, Australia; Mucpharm Pty. Ltd., Kogarah, NSW, Australia

**Keywords:** pseudomyxoma peritonei, shear wave elastography, mucin

## Abstract

**Objectives:**

Pseudomyxoma peritonei (PMP) is a rare peritoneal malignancy characterised by progressive accumulation of mucin within the abdomen. The gold standard of PMP treatment is cytoreductive surgery with hyperthermic intraperitoneal chemotherapy (CRS-HIPEC). BromAc^®^ is a mucolytic therapy developed for patients with inoperable PMP but its efficacy is reduced with hard mucin. Shear wave elastography (SWE) is an ultrasound technique that quantifies the mechanical and elastic properties of tissues. It was hypothesised that SWE could provide a preoperative, objective assessment of mucin consistency.

**Methods:**

A retrospective pilot study of patients with PMP who underwent SWE prior to CRS-HIPEC was conducted. All patients underwent elastography at a specialised radiology practice. SWE results across the four quadrants of the abdomen were compared with intraoperative assessment of mucin consistency.

**Results:**

The study yielded 12 eligible patients between Jan 2020 to Apr 2025. The mean SWE value for hard mucin was 5.9 kPa compared with 4.5 kPa for soft. This difference was not statistically significant (p=0.64).

**Conclusions:**

There is a clinical need for an objective assessment of mucin consistency in patients with PMP. While limited by its small sample size, this study found that the mean SWE result for hard mucin was slightly higher than for soft.

## Introduction

Pseudomyxoma peritonei (PMP) is a rare peritoneal malignancy that is characterised by progressive accumulation of mucinous tumour and fluid within the abdomen [1]. It is most commonly associated with mucinous appendiceal tumours, but can also arise from mucinous tumours of the gastrointestinal tract and ovaries [1], 2]. Mucin from pseudomyxoma can be categorised as soft, semi-hard, and hard with varying physical and chemical differences [1]. Hard mucin has a higher cellular content, water content, protein and lipid concentration and differing thiol distribution pattern than soft mucin; this is thought to account for the differences in texture and hardness [1].

The current standard of care for suitable patients with PMP is curative intent cytoreductive surgery with hyperthermic intraperitoneal chemotherapy (CRS-HIPEC) [3]. Untreated, the accumulation of intraperitoneal mucin leads to increasing abdominal pain and distension, and progressive early satiety and malnourishment secondary to compression and obstruction of the gastrointestinal tract [1], [2], [3]. However, not all patients with PMP are suitable for CRS and technical difficulty and complication rates increase with number of operations [2]. Systemic chemotherapy has limited utility in these patients with an overall response rate of only 20 % [2].

BromAc^®^ is a therapy that has been developed for use in unresectable PMP or surgically unfit patients [4], 5]. A combination of bromelain, a proteolytic enzyme derived from pineapples, and N-acetylcysteine, BromAc^®^ dissolves PMP mucin, facilitating its extraction and assisting with symptom control [4], [5], [6]. It is administered via radiologically or surgically inserted drains into the abdomen [4]. After 24 h, the drains are aspirated with the goal of removing the dissolved mucin [4]. In the *in vitro* setting, BromAc^®^ has been shown to be 100 % effective in dissolving soft mucin, however, this effect is reduced to only 40 % in hard mucin [1].

Currently, the distinction between hard and soft mucin can only be made intra-operatively, based on the subjective assessment of the operating surgeon. Shear wave elastography (SWE) is an ultrasound imaging technique that quantifies the mechanical and elastic properties of tissues [7]. The acoustic radiation force pulse sequence of SWE generates shear waves, the distribution of which provides an absolute measure of the elastic properties of the tissue being assessed [7]. Higher shear wave values correlate with stiffer, more contracted tissues [7]. It is therefore hypothesised that, given the physical differences between hard and soft mucin, SWE could provide an objective measure to make this distinction pre-operatively. SWE could therefore provide additional preoperative information and assist in decision-making when evaluating the risk vs. benefit of BromAc^®^ or other therapies for the management of inoperable PMP.

It remains unclear whether there is a difference in the outcomes of CRS-HIPEC between PMP patients with hard vs. soft mucin. In this case, SWE could also help predict surgical outcomes, guide operative planning and therefore, better counsel higher risk patients.

## Materials and methods

### Study objectives

The primary objective of this study was to establish preliminary support for the hypothesis that SWE can accurately distinguish hard from soft mucin in adults with pseudomyxoma peritonei. In doing so, feasibility for larger scale studies to address this hypothesis could then be assessed.

Secondary objectives included: determining if the intra-operative findings of consistency of PMP are associated with completeness of cytoreduction and impact of hard vs. soft mucin on 30 day post-operative morbidity and mortality.

### Study design

This was a retrospective pilot study of patients with PMP who underwent SWE at Glenn and Partners Radiology (Kogarah, Sydney, Australia) and subsequently had CRS at St George Hospital, Sydney, Australia. SWE was carried out at one institution by one qualified sonographer using a Phillips ultrasound machine with a curvilinear probe. The stiffness of mucin was measured in kilopascals (kPa) and a value was obtained for each of the four quadrants of the abdomen.

Inclusion criteria were: adult patients aged>18 years old with PMP of any origin undergoing CRS between 01/01/2020–30/04/2025 with documented preoperative SWE and intraoperative assessment of hard/soft mucin in each of the four quadrants of the abdomen. The intraoperative assessment of mucin consistency was subjectively determined by one of three consultants responsible for these cases with hard mucin being classified as more solid in form and soft mucin more gelatinous. Patients were excluded if preoperative SWE data was not accessible via the hospital electronic medical record (eMR) or intraoperative findings were not recorded.

The patients were identified from St George Hospital eMR using coding data (“C786 Secondary malignant neoplasm of retroperitoneum and peritoneum”, and “M84806 Pseudomyxoma peritonei”) within the study timeframe. Data collected included: patient age, primary cancer diagnosis, SWE results and intraoperative assessment by the principal surgeon as to whether mucin was “hard” or “soft” for each of the four quadrants of the abdomen. Secondary data points included: peritoneal cancer index (PCI), completeness of cytoreduction score (CCS), operative time, and 30-day mortality and morbidity. The 30-day morbidity and mortality outcomes were: death, unplanned return to theatre, unplanned ICU stay, significant near misses, and unplanned readmissions.

**Figure 1:**
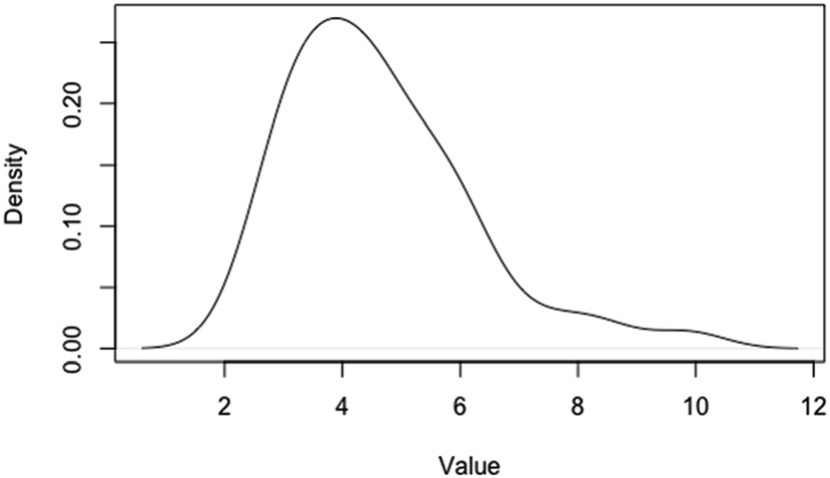
Density plot to assess for normality of data demonstrates that data is positively skewed.

Data was then analysed in RStudio using R(v4.5.0). The normality of the data was interrogated and the data was found to be positively skewed (Figure 1). Given the four data points for each patient were not independent observations, it was acknowledged that clustering could skew the results of the study. Data was accordingly analysed using a generalised linear mixed model with gamma regression to account for both the positively skewed data as well as the clustering effect. As the sample size of this study was small and results were thus unlikely to be statistically significant, the goal of this analysis was to establish baseline data that could then be used to calculate an estimate sample size to adequately power a future prospective study. Calculating this sample size would provide an indication of the feasibility and futility of future studies. PMP is a rare condition and so for a prospective study to be carried out within a reasonable timeframe, this projected sample size would likely need to be less than 100 patients. A larger sample size would either indicate that this method is not feasible to pursue or needs to be conducted as a multi-centre study in order to recruit the necessary number of patients.

This study was approved by the South Eastern Sydney Local Health District Human Research Ethics Committee (HREC 2025/ETH00536). A waiver of consent was sought and obtained on the basis that the study posed no more than low risk to the patient and National Health and Medical Research Council National Statement criteria were met [8]. De-identified data was collected from St George Hospital eMR, stored using REDcap data management software and housed on local hospital servers. Data will be retained for 5 years from the date of publication after which time the data will be deleted.

## Results

16 of the 191 patients who underwent CRS-HIPEC for PMP within the study timeframe had undergone a preoperative SWE. 4 of these patients were excluded due to incomplete data. Of the 12 remaining patients, 10 patients had soft mucin in all quadrants and two patients had mixed hard and soft mucin identified intraoperatively (Tables 1 and 2). Basic demographics, histopathology, PCI and complete cytoreduction (CC) scores are outlined in Table 3. Data points were counted discretely i.e. if a patient only had a description of mucin in the right upper quadrant, only the right upper quadrant shear wave value was counted. As such, there were a total of 48 discrete data points for analysis; three of which relate to hard mucin, the remainder relating to soft mucin.

**Table 1:** SWE value by quadrant.

Case number	SWE, kPa			
	Left upper quadrant	Right upper quadrant	Left lower quadrant	Right lower quadrant
1	6.18	6.4	6.02	7.13
2	2.76	3.45	3.41	4.47
3	2.65	3.04	4.61	5.48
4	4.39	3.62	4.39	4.01
5	2.98	3.5	4.25	4.39
6	4.35	3.3	4.1	3.53
7	2.70	3.32	2.98	3.65
8	3.07	3.64	2.46	3.86
9	7.88	6.22	9.87	4.72
10	5.56	5.36	8.47	3.96
11	5.70	4.64	5.58	4.64
12	4.97	5.13	5.95	5.16

**Table 2:** Intraoperative characterisation of mucin by quadrant (where s=soft, h=hard).

Case number	Mucin			
	Left upper quadrant	Right upper quadrant	Left lower quadrant	Right lower quadrant
1	s	s	s	s
2	s	s	s	s
3	s	s	s	s
4	s	s	s	s
5	s	s	s	s
6	s	s	s	s
7	s	s	s	s
8	s	h	s	s
9	h	h	s	s
10	s	s	s	s
11	s	s	s	s
12	s	s	s	s

**Table 3:** Basic demographics and operation outcomes.

Case number	Age	Sex	Histopathology	PCI	CC
1	69	Female	Low grade mucinous carcinoma peritonei	32	0
2	66	Male	Low grade appendiceal mucinous neoplasm	27	0
3	61	Female	Low grade appendiceal mucinous neoplasm with acellular mucin	16	0
4	56	Male	Low grade appendiceal mucinous neoplasm	28	1
5	40	Male	Low grade appendiceal mucinous neoplasm with acellular mucin	14	0
6	84	Female	Low grade appendiceal mucinous neoplasm with acellular mucin	39	1
7	42	Female	High grade appendiceal mucinous carcinoma peritonei	31	1
8	35	Male	High grade appendiceal mucinous carcinoma peritonei	39	0
9	59	Female	Low grade appendiceal mucinous neoplasm	29	0
10	64	Female	Low grade appendiceal mucinous neoplasm	29	0
11	80	Male	High grade appendiceal mucinous carcinoma peritonei	39	1
12	40	Male	Low grade appendiceal mucinous neoplasm	18	1

**Figure 2:**
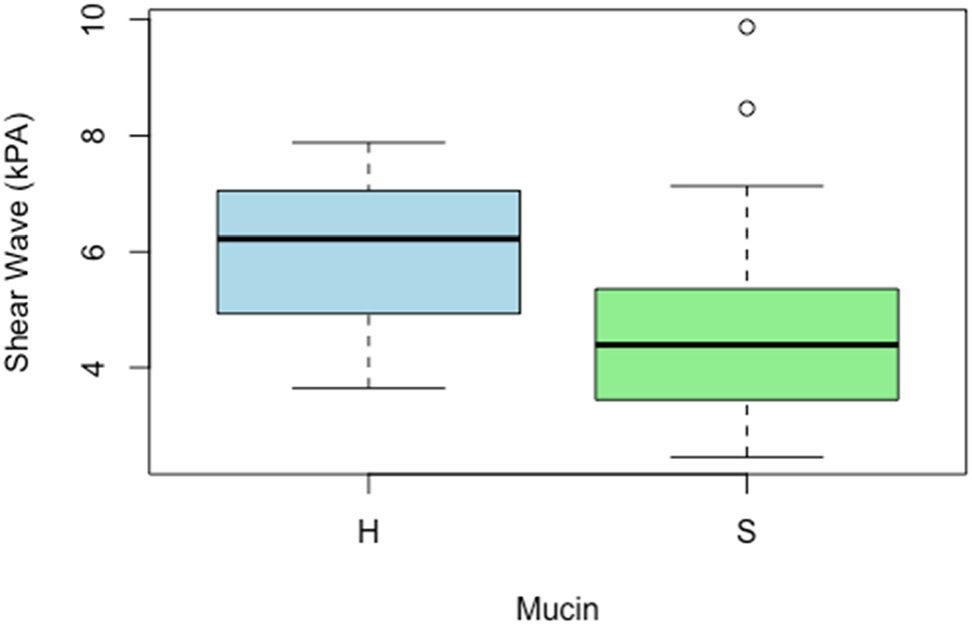
Boxplot comparing shear wave values in kPa of hard (H) and soft (S) mucin.

Hard mucin had a higher mean shear wave value of 5.9 kPa compared to soft mucin, which had a mean value of 4.5 kPa (Figure 2). This was not statistically significant (p-value=0.64).

Secondary outcomes were not evaluated due to the lack of data pertaining to patients with hard mucin compared to soft.

## Discussion

The overall findings from this study support the hypothesis that higher shear wave values may correlate with intraoperatively assessed hard mucin. The findings were not statistically significant however this was not unexpected given the small sample size of the study. In particular, of the 48 quadrants assessed across the 12 patients in this study, only three had hard mucin. This thereby limits the ability to generate a meaningful comparison between the two groups.

PMP is a disease that affects 1–2 per one million people worldwide [9]. In an attempt to maximise the sample size and ensure all eligible patients were captured within this retrospective review, data was collected from 2020 to present. However, the sample size was further limited as SWE was not conducted routinely prior to 2023 and four patients had missing data that prevented inclusion in the data analysis.

Another contributing factor that could be impeding the accuracy of SWE in differentiating hard and soft mucin is the presence of fibrotic tissue alongside the mucin. Inflammation related to PMP, as well as scar tissue from previous CRS, results in fibrosis within the abdomen independent of the hardness of the mucin [9]. Fibrous tissue can trap mucin and result in dense tumour [9]. These denser tumours would result in higher shear wave values even if the mucin trapped within it is soft.

Additionally, the hardness of mucin and its location intraoperatively was recorded as per the PCI, dividing the abdomen into 13 regions, including the mobile small bowel into upper and lower jejunum and ileum. SWE records values only for each of the four quadrants of the abdomen, thus making it difficult to correlate with and analyse against intraoperative findings. Collection bias must also be considered as the hardness of mucin, determined subjectively, was recorded by different surgeons across the patient database.

### Future directions

A prospective study with a larger sample size, and appropriately blinded dual surgeon intraoperative assessment of hard vs. soft mucin would provide valuable information. Based on the 6 % difference between SWE values for hard and soft mucin found in this study, in order to observe a statistically significant difference and provide adequate power to a future study (power=80 %), a sample size of approximately 54 patients would be required. Given the 191 patients who underwent CRS-HIPEC for PMP in the last 5 years, it would be feasible to conduct a prospective study over a relatively short timeframe of approximately 2–3 years in order to appropriately answer this hypothesis.

Additionally, fibroblast activation protein inhibitor (FAPI) PET uses gallium-68 and is most useful in detecting tumours with a strong desmoplastic reaction, such as peritoneal carcinomatosis, as well as non-malignant conditions such as Crohn’s disease and rheumatoid arthritis [10]. The tumour microenvironment of PMP includes an accumulation of immune cells such as tumour-associated fibroblasts [11]. As the cellular content of mucin increases with its hardness, FAPI PET may be able to differentiate hard from soft mucin [1]. Currently, FAPI PET is only available for research purposes in Australia. Future studies could assess its role as an alternative imaging modality in assessing mucin hardness and provide further evidence to support its transition to clinical use [12].

Finally, delayed phase CT is being evaluated for its efficacy in distinguishing the grade of PMP [13]. In contrast to arterial and venous phases, delayed phase CT allows optimal visualisation of PMP lesions as a result of progressive contrast retention, allowing fibrovascular tumour stroma to be distinguished from hypodense mucin [12]. Bai et al. 2025 found that a mixed model combining these radiological findings with clinical biomarkers was effective in identifying the grade of PMP non-invasively [13].

## Conclusions

There is a clinical need for a validated, non-invasive means to differentiate hard from soft mucin in patients with inoperable PMP. At present, SWE cannot be used to predict the efficacy of BromAc^®^ in patients with inoperable PMP. However, in this pilot study SWE values for hard mucin were found to be slightly higher than for soft mucin, supporting the hypothesis that SWE could potentially be utilised to differentiate between hard and soft mucin in a non-invasive manner. While the findings were not statistically significant, this is not unexpected in the setting of the small sample size and limited number of hard mucin data points. Future prospective studies with a larger sample size, and potentially also assessing alternative imaging modalities, should be considered.
